# Work-Related Eye Injuries: A Relevant Health Problem. Main Epidemiological Data from a Highly-Industrialized Area of Northern Italy

**DOI:** 10.3390/ijerph14060604

**Published:** 2017-06-06

**Authors:** Fabriziomaria Gobba, Enrico Dall’Olio, Alberto Modenese, Michele De Maria, Luca Campi, Gian Maria Cavallini

**Affiliations:** 1 Chair of Occupational Medicine, Department of Biomedical, Metabolic and Neural Sciences, University of Modena & Reggio Emilia, 41125 Modena, Italy; dallolio.e@gmail.com (E.D.O.); albertomodenese1@gmail.com (A.M.); 2 Institute of Ophthalmology, University of Modena & Reggio Emilia, 41124 Modena, Italy; michele.demaria86@gmail.com (M.D.M.); campi.luca@policlinico.mo.it (L.C.); gianmaria.cavallini@unimore.it (G.M.C.)

**Keywords:** work-related eye injury, ophthalmological emergency, occupational accident prevention

## Abstract

The province of Modena is one of the most industrialized areas of Northern Italy. The medical records of the Ophthalmological Emergency Department (OED) of Modena University Hospital were studied: there were 13,470 OED accesses in 2014 and in 754 cases that an occupational eye injury occurred. The frequency of work-related eye injuries (3‰) was lower compared to other published studies, but the absolute number is still relevant, showing the need for more adequate prevention, especially in metal work, construction work, and agriculture, where the worst prognoses were observed. Intervention programs must be implemented as early as possible in the working life, considering that the frequency in younger workers is about double that of the oldest age class (3.5‰ vs. 1.8‰), and special attention should also be given to foreigners, who have a 50% higher injury risk. Furthermore, the planning of specific interventions for eye-injured workers may be useful, considering that a previous injury does not appear to encourage the adoption of preventive interventions, and a subgroup of eye-injured workers have a potential risk for new injuries. Finally, the data presented here indicates how OED records, integrated with specific occupational information, can be applied for studies on work-related eye injuries.

## 1. Introduction

Eye injuries represent a frequent cause of emergency ophthalmic visits, and have significant impact in terms of suffering, impairment of life quality, and the reduction work ability. Furthermore, eye injuries pose a relevant cost for the health system and result in a relevant loss of working days worldwide [1]. Work is a recognized and frequent cause of eye injury, representing a proportion ranging from 30% to 70% of the accesses to the Ophthalmological Emergency Departments (OEDs) according to various studies [1,2,3]. As most, if not all, work-related eye injuries can be considered to be preventable [4,5], the development of improved and more stringent preventive measures is both important and urgent, and has a potentially relevant impact. A better and updated knowledge on the epidemiology of work-related eye injuries is of great relevance for the development of new and more effective preventive measures, but, at least in Italy, data on work related eye injuries are scant and insufficiently detailed [6,7].

We present the results of a study on occupation-related ocular injuries in the province of Modena, one of the most densely populated and industrialized areas of Northern Italy, with a high number of active workers, and characterized by a proportion between workers engaged in industrial activities, services and agriculture quite different from the Italian national ratio: 41.9%, 55.6%, and 2.5% in industrial sector, services and agriculture, respectively, vs. 27.3%, 69.1%, and 3.6% in Italy [8]. The risk of work-related eye injuries varies largely, depending on the specific work activity. In order to develop a better knowledge of the phenomenon, a good knowledge of sectors which have a higher burden of specific injury types and the circumstances that are involved are important. For these reasons, using the medical reports of the University Hospital OED, we have evaluated the frequency, type, and other relevant characteristics of ocular injuries which occurred in workers engaged in different sectors. The main results are presented here.

## 2. Materials and Methods

Relevant demographic data, including data on the work-force in Modena province (Italy) were obtained from the local Chamber of Commerce [8] and from the Italian Compensation Authority (INAIL) [9]. In 2014, the number of inhabitants of the city of Modena and the wider province was 701,641. The number of active workers was about 260,000, with approximately 80% Italians and 20% foreign workers [8,9].

In Italy, all persons manifesting acute eye problems of any type are usually referred to a specialized ophthalmology department. For the city and wider area of Modena the only facility is the 24-h Ophthalmological Emergency Department (OED) at Modena University Hospital. Accordingly, we can reasonably assume that the majority (if not all) of the acute eye injuries deserving medical treatment in this area are referred to this OED. A medical report is compiled for all patients arriving at the OED. In the case of an occupational eye injury, a specific section of the medical report is completed, and aside from personal data, the name of the company, the sector, some details of the accident (specific activity performed, causal agent, day, time, and place of the injury), and the main clinical findings (mainly diagnosis, treatment, and prognosis) are collected. In Italy, notified occupational eye injuries are compensated, so workers have an interest to declare the occupational origin of the injury. As such, the data presented here can be considered as adequately representative of the phenomenon of occupational ocular injuries in the province of Modena.

All of the cases of occupational eye injuries recorded in the archive of medical reports of the OED during the period 1 January–31 December 2014 were collected and analyzed. We classified all of the companies of injured workers according to the Italian classification of economic activities “Attività Economiche” (ATECO), i.e., the classification adopted by the Istituto Nazionale di Statistica (ISTAT) for use in economic statistical returns. This classification enabled us to compare the number of eye injuries observed in each ATECO sector with the total number of workers in the same sector in the province of Modena. Statistical quantitative analysis was performed using the Statistical Package for Social Science (SPSS) software, version 23.0 (IBM, Armonk, NY, USA). The study was conducted according to the principles of the Declaration of Helsinki, and the study protocol was approved by the Modena Ethics Committee (record number 292/15, 23 March 2016).

## 3. Results

According to the medical reports of the OED, in the period 1 January–31 December 2014 the total number of visits was 13,470, and in 754 cases (5.6%) the observed lesions were classified as being related to the occupational activity of the patient. In relation to the population of Modena province, the frequency of accesses to the OED due to an acute eye problem (of any type, including e.g., acute bacterial conjunctivitis and acute glaucoma), was 1920 per 100,000 persons. Specifically considering visits related to work activities and the number of active workers in the region in 2014, the annual frequency of occupational eye injury was 3‰.

**Figure 1:**
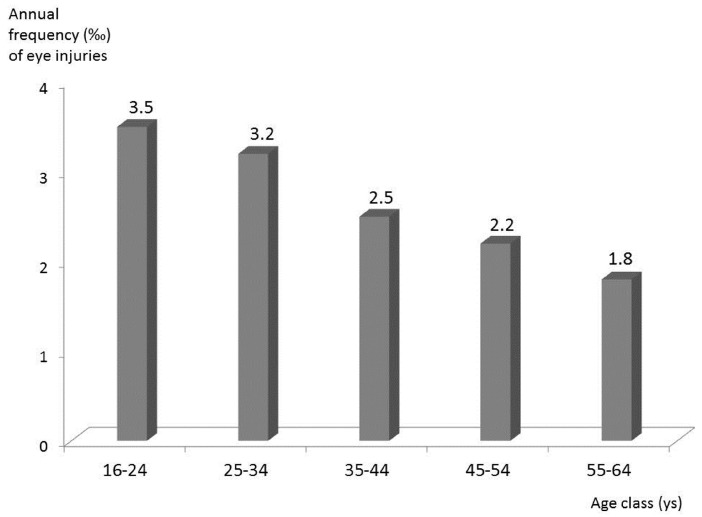
Annual frequency of work-related eye injuries per 1000 workers employed in 2014 in the province of Modena, considering their age classes.

The large majority of work-related injuries occurred in males (87%), with a male/female ratio of 7:1, and was unilateral in presentation: left eye—360 cases, right eye—351 cases. Only 43 cases involved bilateral lesions, and these were essentially all due to contact with chemical agents. The number of subjects involved was lower than the absolute number of OED accesses (respectively n = 716 vs. 754), and this reflected the fact that 28 workers suffered more than one occupational eye injury during the same year. Regarding nationality, 73% of injuries occurred in Italian workers (n = 552), while 27% (n = 203) occurred in foreign workers: considering workforce composition, foreign workers had approximately a 50% higher risk of eye injuries when compared to Italian workers. The mean age of injured workers was 42 years old (Standard Deviation = 12). The frequency of injuries in different age groups is represented in Figure 1. It decreases progressively with age and in 16–24 year-old workers the frequency is about double compared to the 55–64 year-old class (3.5‰ vs. 1.8‰).

**Figure 2:**
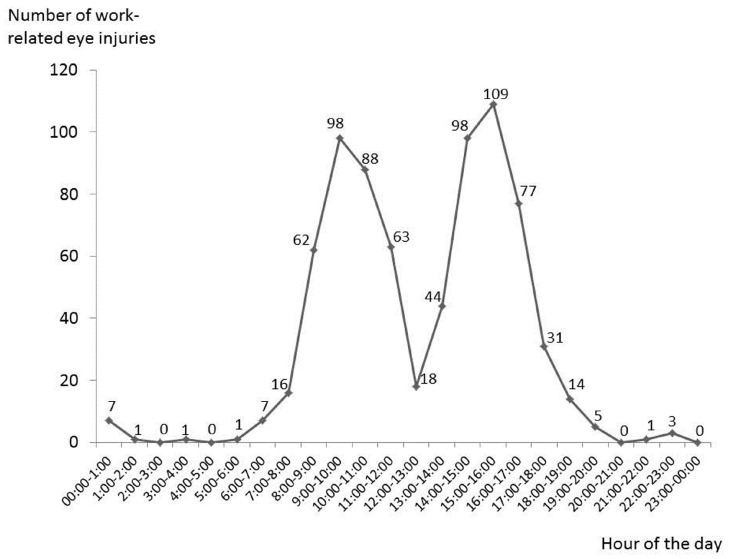
Distribution of work-related eye injuries in 2014 in the province of Modena according to the hour of access to the Ophthalmological Emergency Department (OED).

**Figure 3:**
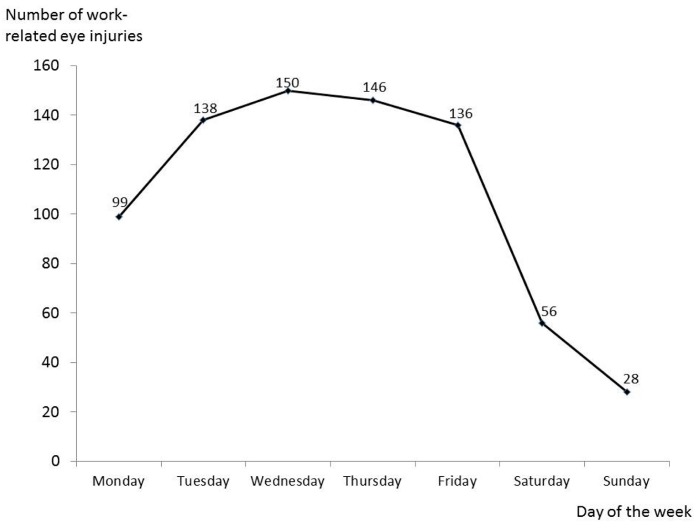
Distribution of work-related eye injuries in 2014 in the province of Modena according to the day of access to the Ophthalmological Emergency Department (OED).

The large majority of injuries (about half) occurred during the central hours of the morning and afternoon: 9–11 and 14–16, respectively (Figure 2). The frequency of injuries from Tuesday to Friday was mainly similar, but was lower on Monday. Less injuries occurred on Saturday and Sunday, but the total number of workers at work during these days is substantially lower when compared to the other days of the week (Figure 3).

**Figure 4:**
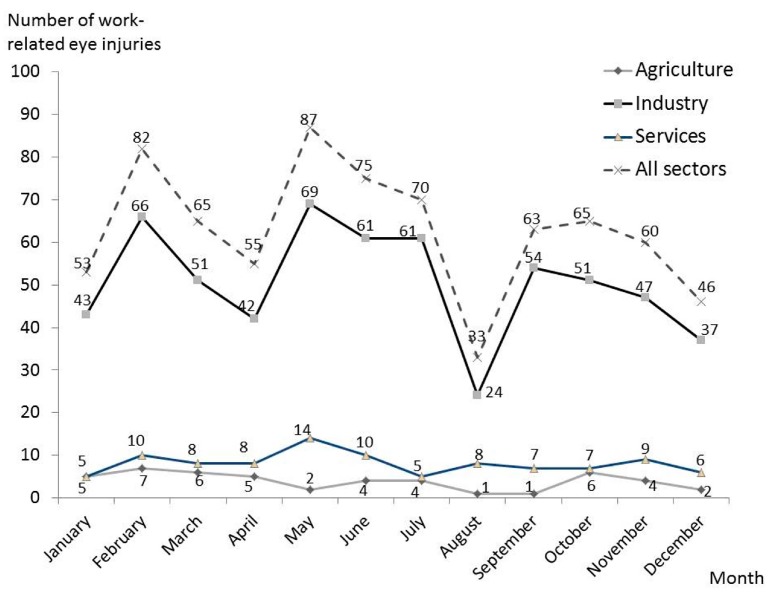
Number of work-related eye injuries in 2014 in the province of Modena in agriculture, industry and the services sectors, according to the month of access to the Ophthalmological Emergency Department (OED).

Considering the distribution of injuries in the different months of the year, the lowest number occurred in August, followed by December, January, and then April. During these months, due to different holiday periods, the number of working days in Italy is lower. Quite interestingly, about one quarter of all injuries occurred in only two months: February (11%) and May (12%) (Figure 4).

This trend suggested an analysis of the injuries which occurred in the different sectors: industry (including construction), agriculture, and services. The results are presented in Figure 4: in agriculture, the highest number occurred in February, while both in industry and services, the highest number occurred during May. The total number of events observed was too low for a more detailed evaluation, e.g., according to the causal agent.

Considering the distribution of the total of work-related ocular injuries (n = 754), 80% (n = 606) occurred in industry, 13% (n = 98) in services, and 6% (n = 47) in agriculture. In relation to the number of workers engaged in these sectors in the province of Modena in 2014 [8], the annual frequency of eye injuries per 1000 workers was calculated: thus, industry is confirmed as the activity with the highest risk (4.9‰), with agriculture slightly lower (3.5‰), and services substantially lower (0.8‰) (Table 1).

**Figure 5:**
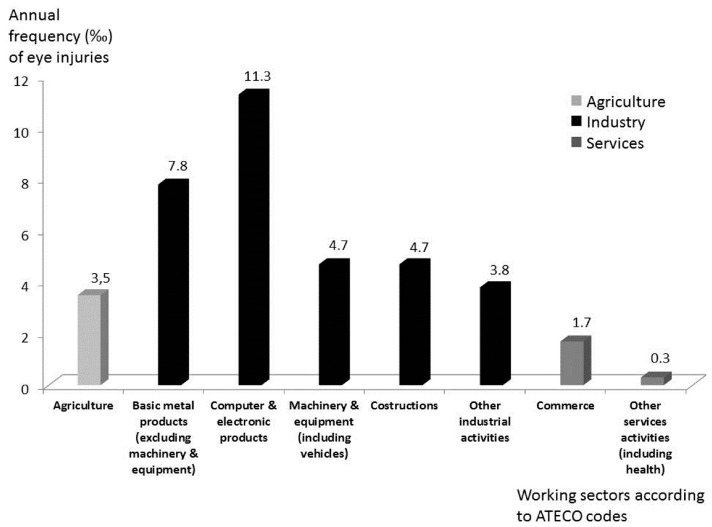
The annual frequency of work-related eye-injuries per 1000 workers employed in 2014 in the province of Modena, considering the main working sectors classified according to the “Attività Economiche” (ATECO) classification codes.

The absolute number of injuries sustained in industry was higher than agriculture and services. According to the “ATECO classification of economic activities”, for the industry sector four groups of activities were classified; activities representing less than the 3% of the total number of employed were merged into a single group (“Other industrial activities”). Due to the high number of ATECO codes included, and due to the spread of injuries, a similar detailed analysis was not able to be achieved in agriculture and all of the ATECO codes were merged. In regard to services, the ATECO codes and the number of injuries collected enabled us to classify the activities into only two groups: “trade including repair” and “other service activities, including health”. The results are presented in Figure 5 and Table 1.

Considering the specific company activity of injured workers and classified according to the abovementioned ATECO codes, the absolute number and frequency of eye-injuries presented in Figure 5 and Table 1 confirm that metal working (including the assembling of various appliances) is the activity inducing the highest risk, followed by construction and agriculture.

In a further analysis we considered the type of lesions that occurred and their distribution among different activities. According to the available descriptions recorded in the medical reports of the OED, the following classification of the causal agents were possible:

- Lesions related to traumatic agents (including foreign body and blunt object trauma);
- Lesions related to chemical agents (with further classification of “alkali”, “acids”, and “detergents”);
- Lesion due to physical agents (mainly electrocution and acute exposures to optical radiation); and
- Splashes of blood or other potentially-infected materials

The results are presented in Table 2: the large majority of injuries were due to a trauma (n = 679, 90% of all occupational injuries). The number of lesions related to chemicals were 61 (8% of all injuries), mainly acid and alkali detergents (25%). Physical agents and biological splashes caused five injuries (about 1% of total occupational eye injuries), and in four cases (<1%) the causal agent was not recorded. We further subdivided the causal agents according to the sector of the company (Table 2). Trauma represents by far the leading cause of injury in all sectors: 80% in services, 92% in industry, and 96% in agriculture. Chemicals were the second cause, and while they represented only 4% in agriculture and 6% in industry, this rose to 15% in services where the chemicals involved were mainly detergents. Injuries related to physical agents were only observed in industry, and biological splashes of potentially infected materials occurred only in services (amongst hospital and laboratory personnel).

**Figure 6:**
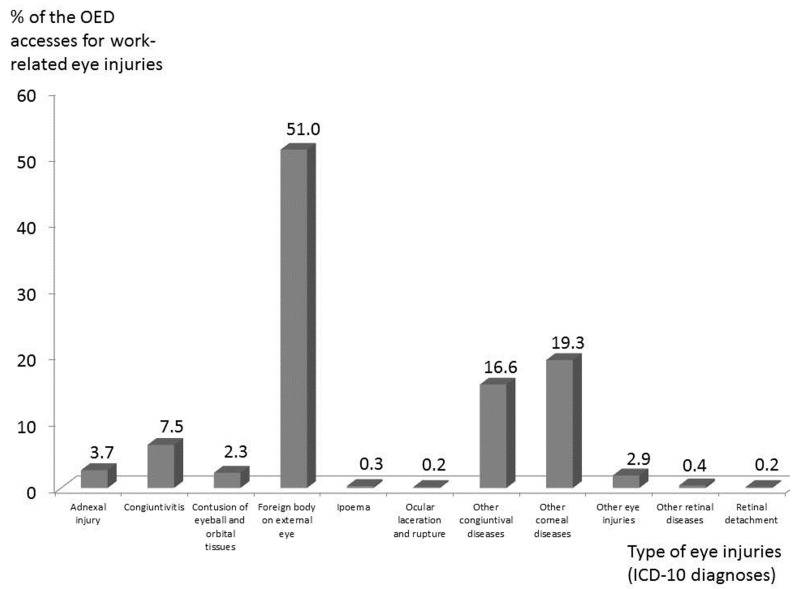
Types of eye injuries classified according to the 10th revision of the International Statistical Classification of Diseases and Related Health Problems (ICD-10) on the total of the 2014 accesses at the Ophthalmological Emergency Department (OED) of the province of Modena for work-related eye injuries.

The main types of ocular injuries classified according to the 10th revision of the International Statistical Classification of Diseases and Related Health Problems (ICD-10) are presented in Figure 6. Regarding medical interventions, the removal of foreign bodies was most common (51% of the cases) (Figure 6). In the whole group, the mean prognosis of the lesion was 2.4 days (Standard Deviation = 1.4), but the longest prognoses were observed in agriculture (3.4 days) compared to industry (2.3 days) and services (2.1 days). The most severe lesion (explosion of the ocular globe resulting in permanent blindness) occurred in agriculture. Of the whole sample, only 29 workers (4%) required a follow-up visit and no further examination and/or treatment was required in the remainder of the cases. An unexpected observation was that 30 workers came back to the OED during the same year. In two cases this was due to a relapse of the lesion, and a further 28 subjects had suffered a new injury (in five workers these injuries numbered more than two). Accordingly, 4% of injured workers accounted for 9% of the accesses to the OED. All of these injuries were trauma lesions due to foreign bodies, and in more than the 90% of cases they occurred in the same eye. For the most part, the workers involved were from metal working, and excluding the mean age (which was slightly higher: 45 vs. 42 years old; *p* < 0.01) all of the other demographic characteristics did not significantly differ from the whole group of injured workers (e.g., nationality, gender, distribution, etc.) and the prognosis was similar (2.5 vs. 2.3 days).

## 4. Discussion

This study is based on a relatively large number of OED accesses (n = 13,470) and work-related injuries (n = 754) compared to other published studies. Nevertheless a reliable comparison of the results is limited by the low number of similar studies published and by various differences, e.g., on the data collected, the mode of collection, the classification of injuries, etc. As an example, other authors include only eye injuries, while we considered all of the accesses to the OED including, e.g., acute glaucoma. Additionally, the objectives of the studies may vary, and whilst our main scope was to obtain indications for a more targeted prevention of work-related eye injuries in workers, others have been more interested in the causes or specific type of injury, on the outcomes of treatments, etc.

With these premises, especially considering the larger number of OED accesses due to our broader inclusion criteria, the annual frequency of eye injuries observed in this study (1920 per 100,000 persons) can be considered coherent with data of other authors, ranging from 310 to 2090 per 100,000 person-years [10,11,12]. In a similar Italian study, a slightly lower frequency of 1131.9 cases per 100,000 person-years was observed [6].

Work-related injuries resulted in 5.6% of all OED accesses: this is lower compared to most other studies, usually ranging from 30% to 70% [1,2,6,13,14,15]. The most likely reason for such a difference is the inclusion in our study of all OED accesses, not limited to trauma (even though most of the work-related accesses were related to traumatic events). Another possible reason is that about half of the workforce in the province of Modena is from the services sector, which has the lowest frequency of eye injury (approximately 20% ≤ industry and agriculture). The possibility of an under-notification cannot be ruled out, but in Italy, occupational eye injuries are compensated, so workers are usually interested in registering a correct notification. However, even if an underestimation cannot be excluded, data suggest a lower risk of occupational eye injuries in workers of the province of Modena compared to some other areas.

Regardless of these differences, we have to observe that the absolute number of work-related injuries (>750/year) is far from negligible. The large majority was unilateral, with a similar proportion of right/left eye involvement: this is in agreement with results of other studies [2,6]. Male workers are most frequently involved (87% vs. 13% in women), as previously reported [2,16,17]. This is not unexpected, considering the larger proportion of male workers traditionally engaged in high risk activities. The mean age of injured workers was 42 years old, which is relatively high compared to other studies [1,2]. However, the frequency in younger workers (16–24 years old) is about double that of the oldest age class (55–64 years old) (Figure 1), suggesting a significant protective role of experience, and this observation is supported by previous results [5,15,18,19].

The distribution of accidents throughout the year (Figure 4) deserves some consideration. The lower frequency observed in December/January is similar to data reported by Cai et al. [2], and is probably related to the lower number of working days due to holidays. This is also the case in August, but no obvious explanation is available for the highest frequencies observed in February and May. The daily time distribution shows two peaks between 9:00 and 10:00 a.m., and between 3:00 and 4:00 p.m. (Figure 2) which does not support any relevant role of fatigue, which has previously been suggested by others [2,16].

Industry (mainly metal work) is the sector with the highest risk of eye injuries, with a frequency of 4.9‰ (absolute number: n = 606). The highest-risk activities were the production of various electrical and electronic appliances, which is a common activity in this region. However, though not unexpectedly, the construction sector also had a high level of risk, followed by agriculture (3.5‰). Additionally, the frequency in services (0.8‰) is about 1/6 of that compared to industry (Figure 5 and Table 1). This distribution is substantially in agreement with the results of other studies, e.g., in a recent study conducted in the Southwest Region of China [2].

In industry, trauma was by large the most prevalent type of injury, and foreign bodies and blunt object trauma were the most frequent causes (70% vs. 20%, respectively: Table 2 and Figure 6). In agreement with various other studies, grinding, soldering, and compressed air use were, by large, the most frequently reported activities performed by workers, even if a detailed description of the specific activity was available only in part of the reports. The industry sector is also the only one with injuries related to physical agents (and included all of the cases of electrocution), but the overall number is really low (five cases, see Table 2). No cases of eye injuries due to optical radiation, a relevant occupational risk for acute and chronic adverse eye effects in various occupational activities [20], were observed, which is at variance with the findings of the study of Senriken et al. [16].

Considering agriculture, even if the absolute number of injuries is much lower compared to industry (47 vs. 606, respectively), the frequency is similar (4.9‰ vs. 3.5‰, Table 1), suggesting a similar degree of risk. Almost all injuries sustained in agriculture were related to trauma (45 of 47 cases), and the remaining two cases were due to chemicals (Table 2). The seasonal distribution showed a reduction of injuries in the summer when we expected a higher number of accidents due to the increase of working hours. The reason for this is unclear. Furthermore, in agriculture the prognosis (3.4 days) was about the 50% higher compared to other sectors, and also the most severe eye injuries, inducing the frequency of those causing permanent blindness, occurred in this sector.

The service sector registered the lowest frequency of injuries (0.8‰, about 1/5 of industry), but even so, the absolute number (n = 98) is not small and represents 13% of all injuries (Figure 5 and Table 1). In this sector, the highest proportion of injuries was observed to be caused by chemicals, mostly due to cleaning activities. Furthermore, this sector included five injuries caused by splashes of blood or other biological materials, all sustained by laboratory and hospital workers (Table 2). In this study some specific injuries which have previously been reported in this sector, as bottle cork injuries [21,22], were not observed. The seasonal distribution of injuries in services is similar to industry, but as previously discussed, the reasons for this trend are not obvious, especially considering that the main activities of this sector are rather similar throughout the year (Figure 4).

Considering the diagnosis of eye injuries classified according to ICD-10 (Figure 6), our results are in agreement with another Italian study based on a similar number of annual OED accesses [6], showing that the vast majority of occupational injuries were “closed globe injuries”, representing 98.8% of the total of OED accesses, followed by “open globe injuries” (0.8%) and adnexal injuries (0.3%). In our sample closed globe injuries represented the 96.1% of the total number of OED accesses, open globe, 0.2%, and adnexal injuries, the remaining 3.7%. Comparing the types of the most frequent occupational injuries (closed globe), in our sample a foreign body was detected in 51% of the cases, which is comparable with Fea et al.’s [6] results (60.5%), as is the proportion of corneal and conjunctival injuries, while we found fewer cases of retinal diseases. Finally, Fea et al. found that foreign bodies of the external eye were more significantly represented in work-related injuries than in home injuries. As only data on occupational eye injuries were collected, no comparison with non-occupational injuries was possible; nevertheless, our interest was focused on the risk of work-related injuries, with the aim to evaluate the need of preventive measures and, possibly, to give some suggestions on interventions.

Considering now the severity of the injuries, we have to observe that, in our sample, the proportion of permanent blindness which occurred was consistently lower compared to that reported in other studies including workers engaged in various occupations. Our study raised a frequency of <0.5%, compared to higher reported percentages, ranging from 3% to 10% [4,14,17,18,23,24,25]. Furthermore, the mean prognosis (2.4 ± 1.4 days) was also lower compared to other studies, where values ranging from three days to more than two weeks have been reported [26,27,28]. All of these differences seem rather encouraging, and in conjunction with the lower proportion of work-related accesses to the OED, they support the hypothesis of a lower occupational eye risk occurring in the province of Modena. The reason for this is not obvious, and possibly merits further investigation.

Another relevant result is that 28 workers (approximately 4% of the workers injured in 2014) sustained at least a second injury during the same year, and in more than 90% of cases in the same eye. Five workers had more than two injuries. All of these workers were from the metal-working sector, and the injury was due to metal foreign bodies. Excluding the mean age, which was slightly higher than the overall sample (45 vs. 42 years old; *p* < 0.01), their demographic characteristics did not significantly differ from the main group. The possibility of repeated injuries suggests that, currently, the relevance of work-related injuries, at least of eye injuries, is not adequately acknowledged and, consequently, apparently does not induce in the management the awareness of an issue with the “work safety culture” of the company. As a matter of fact, for an effective prevention, including a reduction of the risk of injuries, a comprehensive improvement of the “work safety culture” is needed. To pursue this objective, the attitudes and beliefs of the workers in perceiving that safety at work is a real value to the company are essential prerequisites. This requires an involvement of different levels of the whole organization, including managers and supervisors [29] while, on the contrary, interventions limited to changing the “work safety climate”, e.g., limited to worker’s training or to the introduction of new protective equipment, are potentially insufficient or, at least, less effective [30].

This study has some limitations. The first is that, being based on medical records collected by the ophthalmologists of the OED during their examinations, the description of specific activities, the correct use (or not) of personal protective equipment (PPE), and other relevant occupational data were not available. Furthermore, in Italy a specific and stringent legislation on the prevention of occupational diseases and injuries and health promotion, based on the EU Directives, is in force: OED records did not allow us to infer any data regarding to the effective respect of this law in the workplaces where injury occurred. Moreover, OED records cannot give us any indication on the culture of safety in the companies, and, accordingly, we could not identify possible cognitive and motivational biases [31], potentially relevant contributors to accidents and to the development of effective preventive measures. The underlying idea in analyzing OED records was the possibility to use available, rather than ad hoc material for a study on the prevention of occupational eye injuries. Another possible limitation of the study is that an underestimation of the total number of occupational eye injuries cannot be excluded. Especially in agriculture and construction, some cases may go unnoticed, but due to the relevance of the organ involved, it is most likely this is only possible for a limited number of minor eye injuries, and considering that, in Italy, occupational injuries are compensated, workers are interested in making a proper notification. Another possibility is that not all of the workers with eye injuries that occurred in the province of Modena accessed the OED of Modena; on the other hand, it is also likely that some of the injuries included in this study occurred in workers transported to the Modena OED from workplaces outside of the province of Modena: this hypothesis is somewhat supported by the observation that about 9% of the companies of the injured workers included in the study are based outside of the Modena province. As a final limitation, the data on the medical costs of these injuries are not available, but would have added an interesting dimension to the study. Regardless of these issues, however, we are confident that these limitations do not bias the principal findings of the study.

## 5. Conclusions

Our results on occupational eye injuries in the Italian region of Emilia Romagna show a lower frequency compared to other studies previously published in other countries and also in Italy, with a relatively shorter prognosis and a much lower proportion of permanent blindness. Nevertheless, the absolute number of occupational eye injuries is still significant, showing that further prevention strategies are needed. We have also to consider that our data is only a look at a one-year trend: further analysis on longer periods, e.g., 5–10 years, should be useful for a deeper knowledge of the risk and of the trends, and to look further at associations.

In planning future prevention strategies, metal work (including the production of electrical and electronic appliances), construction work, and also agriculture where the worst prognoses were observed, should be given a higher priority. The higher frequency of injuries in younger workers supports the need of an implementation of preventive measures as early as possible in working life, and special attention should also be given to foreign workers.

The observation of the noticeable number of re-injuries suggests a current inadequate attention of the companies to the risk. To effectively improve work safety culture an involvement of managers and supervisors is needed, and preventive actions have to be planned at various levels. At the same time, an improvement of work safety climate has also to be considered, e.g., a re-organization of the job tasks following an appropriate analysis of safety problems, and specific training not limited to the workers, but also addressed to supervisors.

As a final note, the data presented here are an example of how medical records routinely recorded during the OED examinations, opportunely integrated with additional information on, e.g., the use of protective devices, can be utilized for studies on work-related eye injuries, and to follow the trends in the phenomenon and the possible impact of preventive measures where they are introduced.

## Figures and Tables

**Table 1:** The number of workers employed in the province of Modena in 2014 in various working sectors classified according to the “Attività Economiche” (ATECO) classification system, number of occupational eye injuries, and the annual frequency per 1000 workers.

Working Sector		Workers Employed	Eye Injuries (Total Number)	Eye Injuries (Frequency ‰)
AGRICULTURE		13,411	47	3.5
INDUSTRY		123,701	606	4.9
Manufacturing	Basic metal products (excluding machinery and equipment)	13,624	106	7.8
	Computer and electronic products	5557	63	11.3
	Machinery and equipment (including vehicles etc.)	25,010	117	4.7
Construction		23,325	109	4.7
Other industrial activities		56,185	211	3.8
SERVICES		122,107	98	0.8
Trade (including repair)		42,215	72	1.7
Other services activities including health services		79,892	26	0.3
ALL SECTORS		259,219	754 ^(1)^	3

^(1)^ In three cases, data on the occupational sector was missing.

**Table 2:** The number of occupational eye-injuries sustained in 2014 in the province of Modena in agriculture, industry, and services, classified according to the causal agent involved (within brackets the percent values are reported).

Working Sector	Physical Agents	Traumatic Agents	Chemical Agents	Biological Splashes
AGRICULTURE (100%)	0 (0%)	45 (95.7%)	2 (4.3%)	0 (0%)
INDUSTRY (100%)	5 (0.8%)	556 (91.7%)	44 (7.3%)	0 (0%)
SERVICES (100%)	0 (0%)	78 (79.6%)	15 (13.3%)	5 (5.1%)
ALL SECTORS (100%)	5 (0.7%)	679 (90.1%)	61 (8.1%)	5 (0.7%)

NB: In four cases (0.5%) the causal agent of the work-related eye injury was not recorded.
